# Risk Assessment After a Severe Hospital-Acquired Infection Associated With Carbapenemase-Producing *Pseudomonas aeruginosa*

**DOI:** 10.1001/jamanetworkopen.2018.7665

**Published:** 2019-02-15

**Authors:** Joost Hopman, Corianne Meijer, Nikki Kenters, Jordy P. M. Coolen, Mohammad R. Ghamati, Shaheen Mehtar, Reinout van Crevel, Wim J. Morshuis, Ad F. T. M. Verhagen, Michel M. van den Heuvel, Andreas Voss, Heiman F. L. Wertheim

**Affiliations:** 1 Radboudumc Center for Infectious Diseases, Department of Medical Microbiology, Radboud University Medical Center, Nijmegen, the Netherlands; 2 Department of Cardiothoracic Surgery, Radboud University Medical Center, Nijmegen, the Netherlands; 3 Academic Unit for Infection Prevention and Control, Department of Interdisciplinary Health Sciences, Faculty of Medicine and Health Sciences, Stellenbosch University, South Africa; 4 Center for Infectious Diseases, Department of Internal Medicine Radboudumc, Radboud University Medical Center, Nijmegen, the Netherlands; 5 Department of Pulmonary Diseases, Radboud University Medical Center, Nijmegen, the Netherlands; 6 Department of Medical Microbiology and Infectious Diseases, Canisius-Wilhelmina Hospital, Nijmegen, the Netherlands

## Abstract

**Importance:**

Resistance of gram-negative bacilli to carbapenems is rapidly emerging worldwide. In 2016, the World Health Organization defined the hospital-built environment as a core component of infection prevention and control programs. The hospital-built environment has recently been reported as a source for outbreaks and sporadic transmission events of carbapenemase-producing gram-negative bacilli from the environment to patients.

**Objective:**

To assess risk after the identification of an unexpected, severe, and lethal hospital-acquired infection caused by carbapenemase-producing *Pseudomonas aeruginosa* in a carbapenemase-low endemic setting.

**Design, Settings, and Participants:**

A case series study in which a risk assessment was performed on all 11 patients admitted to the combined cardiothoracic surgery and pulmonary diseases ward and the hospital-built environment in the Radboud University Medical Center, the Netherlands, in February 2018.

**Exposures:**

Water and aerosols containing carbapenemase-producing (Verona integron-mediated metallo-β-lactamase [VIM]) *P aeruginosa.*

**Main Outcomes and Measures:**

Colonization and/or infection of patients and/or contamination of the environment after the detection of 1 patient infected with carbapenemase-producing (VIM) *P aeruginosa*.

**Results:**

A total of 5 men (age range, 60-84 years) and 6 women (age range, 55-74 years) were admitted to the combined cardiothoracic surgery and pulmonary diseases ward. The risk assessment was performed after carbapenemase-producing (VIM) *P aeruginosa* was unexpectedly detected in a man in his early 60s, who had undergone a left-sided pneumonectomy and adjuvant radiotherapy. No additional cases (colonization or infection) of carbapenemase-producing (VIM) *P aeruginosa* were detected. Plausible transmission of carbapenemase-producing *P aeruginosa* from the hospital environment to the patient via the air was confirmed by whole-genome sequencing, which proved the relation of *Pseudomonas* strains from the patient, the shower drains in 8 patient rooms, 1 sink, and an air sample.

**Conclusions and Relevance:**

This study suggests that rethinking the hospital-built environment, including shower drains and the sewage system, will be crucial for the prevention of severe and potential lethal hospital-acquired infections.

## Key Points

**Question:**

To what extent does the hospital-built environment contribute to the transmission of carbapenemase-producing gram-negative bacilli?

**Findings:**

In this case series study of 11 patients, a clustered localization of 8 shower drains, 1 sink, a positive air sample, and 1 patient were colonized and infected with carbapenemase-producing (Verona integron-mediated metallo-β-lactamase [VIM]) *Pseudomonas aeruginosa.* All identified sites were drained to 1 collection sewage point, likely resulting in airborne transmission originating from shower drains plus extensive contamination of water sources (eg, sinks) in the hospital-built environment.

**Meaning:**

Rethinking the hospital-built environment, including details such as shower drains and the sewage system, will be crucial for the prevention of severe and potential lethal hospital-acquired infections.

## Teaser

This case series study assesses risk after the identification of an unexpected, severe, and lethal hospital-acquired infection caused by carbapenemase-producing *Pseudomonas aeruginosa* in a carbapenemase-low endemic setting in the Netherlands.

## Introduction

The identification of carbapenemase-producing Enterobacteriaceae in environmental samples from New Delhi, India, in 2011 had important clinical implications for people living in low-resource settings who are dependent on public water and sanitation facilities.^1^ To ensure early detection and international surveillance of resistance, it was advised to incorporate environmental sampling as well as examination of clinical isolates in screening strategies.^1,2^ Currently, carbapenem-resistant strains, including *Pseudomonas aeruginosa* and *Acinetobacter baumannii*, are also spreading rapidly to and within high-resource countries.^3,4^ Therefore, the World Health Organization (WHO) has issued measures to control the spread of carbapenemase-producing microorganisms, focusing on Enterobacteriaceae, *P aeruginosa*, and *A baumannii*.^5^ One of the strategies to halt the spread of multidrug-resistant microorganisms is through implementing the WHO infection prevention and control core components, of which the hospital-built environment is added as one of the new core elements.^6^ Sinks are part of this hospital-built environment; the removal of sinks from rooms in the intensive care unit has resulted in the reduction of colonization and infections with multidrug-resistant microorganisms.^7,8,9^ In addition, waste water has been recently identified as a source of transmission of carbapenemase-producing Enterobacteriaceae in an intensive care unit in the United States.^10^ Furthermore, hospital plumbing systems have been revealed as a reservoir of carbapenem resistance by genomic analysis,^11^ and shower faucets have been shown to be important in the level of air contamination in an experimental shower unit.^12^ We report the results of a risk assessment conducted after the identification of an unexpected, severe, and lethal hospital-acquired infection caused by carbapenemase-producing *P aeruginosa* in a carbapenemase-low endemic setting.

## Methods

### Screening

After the identification of a patient who was unexpectedly colonized with a highly resistant microorganism, fellow patients were screened according to Dutch national guidelines.^13^ Rectal and throat swabs of patients at risk were screened for the presence of the highly resistant microorganism. Drain sampling (sinks and shower drains) was performed by inserting the E-Swab (BD) into drain holes. Environmental air sampling (1000 L) was performed using MAS-100 Eco (Lighthouse Worldwide Solutions) directly after the shower had run for 10 minutes. The air sampler was placed on the ground in the middle of the bathroom. Air sampling was repeated 15 minutes after the shower was turned off. All cultures were screened for carbapenemase-producing microorganisms following standard operating procedures by phenotypic analysis (carbapenem inactivation method) and genotypic analysis of all *P aeruginosa* isolates with an E-test for meropenem greater than 2 mg/L.^14^ The screening of patients and environmental sampling was conducted as part of a routine outbreak investigation and therefore no institutional review board approval was requested. Patients underwent screening after oral consent was obtained. Written informed consent was obtained from the relatives of the deceased patient. The article is in line with the guidance provided in the appropriate use and reporting of uncontrolled case series in the medical literature.^15^

Cleaning and disinfection of environmental surfaces of the patient room (bathroom included) was performed by daily cleaning of all surfaces plus disinfection of all high-touch surfaces using Bacillol 30 (Hartman) and Terralin (Schülke & Mayr Benelux). Terminal cleaning and disinfection of all surfaces including the bathroom was performed in case of patient transfer or discharge. Chlorine 0.1% solutions were used to disinfect the stainless steel shower drains and sink drains after mechanical cleaning with a brush.

### Whole-Genome Sequencing

A sequencing library was constructed by using 2- to 5-ng genomic DNA as input for the Nextera XT DNA Library Preparation Kit (Illumina), and paired-end reads of 2 × 150 base pairs were generated using the Illumina NextSeq 500 (Illumina). Reads were filtered and cleaned by applying Trim galore, version 0.4.1,^16^ and an in-house python script. Read coverage was calculated as the total amount of sequenced nucleotides after filtering divided by the length of the reference genome, *P aeruginosa* Carb01 63 (RefSeq:NZ_CP011317.1). Samples with coverage less than 20× were omitted for further analyses. SPAdes, version 3.10.1,^17^ was used to assemble reads to contigs. Multilocus sequence typing types were derived from the contigs using multilocus sequence typing, version 2.5 (https://github.com/tseemann/mlst). Resistance genes were detected by using Abricate (https://github.com/tseemann/abricate) with database Resfinder.^18^ To obtain core single-nucleotide polymorphisms (SNPs), defined as SNPs that are shared among all strains, all samples including a reference set of 10 *P aeruginosa* strains were analyzed using kSNP, version 3.021.^19^ Minimal Spanning Tree was created using Kruskal’s algorithm.^20^ The Radboudumc Service department performed detailed mapping of the sewage systems in the pulmonary and thoracic surgery department.

## Results

In February 2018, we unexpectedly detected carbapenemase-producing (Verona integron-mediated metallo-β-lactamase [VIM]) *P aeruginosa* in a man in his early 60s who had undergone a left-sided pneumonectomy and adjuvant radiotherapy. The patient had received a diagnosis of a pT3N2M0 adenoid cystic carcinoma 29 years prior to admission and was referred to our surgical unit because of a locoregional recurrence in his chest wall. After resection of the dorsal parts of ribs 9, 10, and 11, the patient developed a wound infection, resulting in a fistula to the residual pleural cavity. A Clagett open-window thoracostomy was performed to obtain adequate drainage of the cavity, which was surrounded by heavily calcified fibrous scar tissue. Shortly after the open window was created, the cavity became infected with multidrug-resistant *P aeruginosa*. A wide antibiotic multiresistance profile was observed, with resistance to meropenem, gentamicin sulfate, tobramycin sulfate, and ciprofloxacin hydrochloride and variable activity for ceftazidime sodium and amikacin sulfate. Only piperacillin sodium-tazobactam sodium and colistin sulfate displayed valuable activity against the isolated multidrug-resistant *P aeruginosa*. Antibiotic regimens consisted of combinations with piperacillin-tazobactam, ceftazidime, colistin, and amikacin in combination with local application of acetic acid by packed gauzes that were changed daily. However, the infection persisted, finally leading to an erosion and bleeding from the descending aorta. The aorta was inaccessible for surgical repair and, after the patient was stabilized, a thoracic endovascular aortic repair was performed, covering the defect by means of a stent. However, after 3 days, the patient developed recurrent bleeding, originating from the aorta proximal to the thoracic endovascular aortic repair stent. Extending the thoracic endovascular aortic repair stent was considered, but the patient refused further treatment and died the same day. More important, no risk factors for the carriage of carbapenemase-producing microorganisms (eg, foreign travel) could be identified in our patient, which made us consider alternative routes of transmission of the *P aeruginosa* from other patients or the environment.

### Patient, Environmental, and Air Screening

The unexpected finding of a patient with carbapenemase-producing *P aeruginosa* resulted in screening for colonization in all 11 hospitalized patients admitted to the same ward. Following national guidelines, we initiated the screening of all admitted patients in the same ward at risk for colonization with carbapenemase-producing gram-negative bacilli. All *P aeruginosa* isolates in the ward were analyzed for carbapenemase production. No additional cases (colonization or infection) were detected. Following the national guidelines, no health care professionals were screened for multidrug-resistant gram-negative bacilli. Thorax, urine, and blood cultures obtained in the 7 months between the initial patient’s left-sided pneumonectomy and the open-window thoracostomy were evaluated for the presence of *P aeruginosa*. During this period, 10 wound cultures were obtained, of which 2 wound cultures were positive for *Staphylococcus epidermidis* and *Propionibacterium acnes*; in addition, 1 urine culture was positive for *Citrobacter koseri.* None of the cultures was positive for *P aeruginosa.* In the absence of other patients with positive cultures for *P aeruginosa* and with the knowledge that the patient’s cultures were negative prior to the Clagett open-window thoracostomy, we initiated environmental screening focusing on moist areas of rooms in which our patient had been hospitalized.

For efficiency reasons we conducted environmental sampling in a stepwise approach. In the entire workup we conducted 4 rounds of screening of environmental surfaces (eTable in the Supplement). After finding an identical *P aeruginosa* from a shower drain in the patient’s room, the environmental sampling was extended to the entire ward. One sink that had a negative result for *P aeruginosa* in the first screening had a positive result in the second screening 2 weeks later. After evaluation of the first positive environmental samples we decided to investigate possible airborne transmission from the shower drains by collecting air samples directly after the shower had run for 10 minutes and 15 minutes after the shower had been turned off.

**Figure 1.**
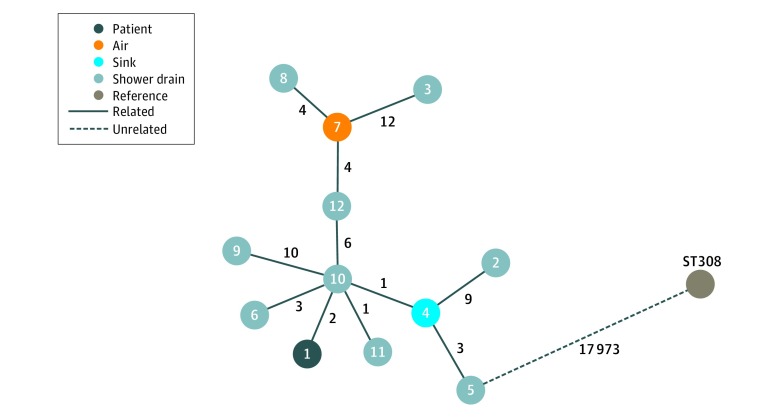
Whole-Genome Sequencing, Minimum Spanning Tree Strains of *Pseudomonas aeruginosa* from the patient, the shower drains, 1 sink, and the air sample are identical with limited single-nucleotide polymorphisms (SNPs; numbers between circles) difference (range, 1-12 SNPs) in comparison with 10 reference strains (≥17 973 SNPs).

**Figure 2.**
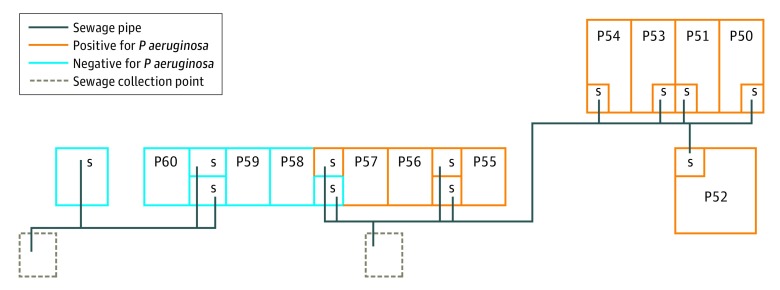
Schematic Ward Map With Sewage Systems and Carbapenemase-Producing *Pseudomonas aeruginosa*–Positive Shower Drains in Patient Rooms Schematic ward map illustrates the clustering of carbapenemase-producing *P aeruginosa*–positive shower drains in patient rooms (P50-P60) and carbapenemase-producing *P aeruginosa*–negative shower drains in patient rooms in relation to the sewage collection points. The patient was admitted to room P54 before undergoing the Clagett open-window thoracostomy; this room had negative results for *P aeruginosa* in the initial environmental screening. After surgery the patient was admitted to room P56, where the shower drain had positive results for *P aeruginosa*. S indicates shower.

A culture of 1 air sample obtained 15 minutes after use of a shower drain with positive results for *P aeruginosa* yielded carbapenemase-producing *P aeruginosa.* Multiple additional different gram-negative bacilli (*Acinetobacter pitti*, *Pseudomonas putida*, *Acinetobacter* spp, and *Enterobacter cloacae)* were found in other air samples after the showers in the initial room and 7 other patient rooms had run. Transmission of carbapenemase-producing *P aeruginosa* between the patient, the air sample, and the hospital environment was confirmed by whole-genome sequencing (Figure 1), which showed that *Pseudomonas* strains from the patient, the shower drains in 8 patient rooms, 1 sink, and the air sample were identical, with limited difference in SNPs (range, 1-12 SNPs) in comparison with reference strains. A schematic overview of the ward layout shows the clustered localization of the shower drains with positive results for *P aeruginosa* in relation to the collection sewage point (Figure 2). After screening all other patients admitted to the ward, the environment, and the air, airborne transmission was considered the most likely source of transmission of the carbapenemase-producing *P aeruginosa* from the shower drain to the pleural cavity accessed by the Clagett open-window thoracostomy. A multidisciplinary infection prevention and control team decided to limit showering for patients with open wounds or severely immunocompromised patients, including those in rooms with—at that time—negative culture results for *P aeruginosa*. All admitted patients were informed about the potential risks and consequences of these new findings.

### Cleaning and Disinfection of Environmental Surfaces

Cleaning and disinfection of surfaces was intensified to a frequency of once daily. Special attention was paid to mechanical and chemical removal of biofilms in shower drains. These measures were initially successful and resulted in environmental samples negative for *P aeruginosa*. After the subsequent air samples remained negative for *P aeruginosa*, we reduced the frequency of cleaning and disinfection to once weekly and finally resumed normal routine environmental cleaning and disinfection practices. However, after we stopped the intensified cleaning and disinfection, shower drains showed regrowth of carbapenemase-producing *P aeruginosa* within 1 week. As a consequence, we reintroduced intensified cleaning and disinfection practices once again in the affected rooms and have explored new interventions with copper and dry-membrane shower drains. None of these interventions have thus far been successful at preventing growth of *P aeruginosa*.

## Discussion

We describe a severe and lethal hospital-acquired infection caused by carbapenemase-producing *P aeruginosa,* likely caused by airborne transmission after shower use. Furthermore, we were able to detect the migration through the shower drain plumbing of the carbapenemase-producing *P aeruginosa* to 7 adjacent patient rooms.

Resistance of gram-negative bacilli to carbapenems is rapidly emerging worldwide and carbapenems are last-resort β-lactam antibiotics needed for the treatment of severe infections. However, in the Netherlands, resistance to carbapenems is still very low, owing to high human and financial resources, resulting in strong national infection prevention and control, surveillance, and antibiotic stewardship programs that are aligned with the guidance from the European Centre for Disease Prevention and Control.^21^

This case illustrates the importance of the hospital-built environment in controlling the spread of carbapenemase-producing microorganisms, as advocated by WHO in the infection prevention and control core components.^6^ In 2016, WHO defined the hospital-built environment as a core component of infection prevention and control programs. Owing to the emergence of resistance of gram-negative microorganisms to antibiotics and disinfectants, it is becoming pivotal to limit the presence of the preferred moist habitat of those bacteria as much as possible in hospitals worldwide.^22,23^ Sinks and hoppers have been reported as sources for outbreaks and sporadic transmission events of gram-negative bacilli from the environment to patients, and interventions to reduce the transmission from these sources to the patients should therefore be prioritized when trying to protect the patient. Removal of a source for carbapenemase-producing bacilli is the most efficient intervention to stop transmission. Despite the fact that a study has shown the efficacy and feasibility of this approach for sinks in an intensive care unit setting,^7^ this is not a likely solution for contaminated shower drains. New engineering solutions should be developed to reduce the risk of aerolization of microorganisms from shower drains.

This investigation was conducted to explain an unexpected carbapenemase-producing *P aeruginosa* in a setting in which carbapenemase-positive microorganisms are rare. We performed a retrospective analysis to investigate the presence of other VIM-positive *P aeruginosa* strains up to 1 year prior to the presented case. We could identify only 1 additional case in the hematology ward. That ward is located on the other side of the hospital (>500 m away) and the *P aeruginosa* was cultured 4 months earlier and never thereafter. We could not detect the initial source for this transmission event; it is unlikely the patient who initially underwent a left-sided pneumonectomy and adjuvant radiotherapy could have been the index patient owing to the thorax, urine, and blood cultures negative for *P aeruginosa* that were obtained in the period between the left-sided pneumonectomy and the open-window thoracostomy. Furthermore, soon after the initial identification of our patient, we confirmed that the shower drains of 7 additional patient rooms were positive for *P aeruginosa* by whole-genome sequencing. It seems unlikely that the carbapenemase-producing *P aeruginosa* could have migrated through biofilms to the other patient rooms in the time between the first positive patient culture and the positive environmental samples. Hand hygiene compliance in the affected ward is routinely measured since the introduction of a hospital hand hygiene campaign 4 years ago and is currently high (80% compliance for the 5 moments of hand hygiene according the WHO). Also, no other surfaces that came into contact with the pleural cavity of the patient (accessed by Clagett open-window thoracostomy) tested positive for *P aeruginosa*. Last, our patient had no known risk factors for colonization with a carbapenemase-producing microorganism.

### Limitations

Some limitations of this study need to be addressed. First and most important, this is a risk assessment after 1 severe case with carbapenemase-producing *P aeruginosa*. No environmental cultures were available prior to the first positive patient culture. Furthermore, we provide data from only 1 hospital; more evidence is needed from other contexts in both high- and low-resource settings.

## Conclusions

Given the substantial effect of infections with carbapenem-producing gram-negative bacilli on the clinical outcome of patients with open wounds, appropriate measures should be taken to reduce the risk of airborne transmission of gram-negative bacilli to open wounds during and after showering. Interventions should focus primarily on hardware (new engineering solutions for showers and plumbing systems). We advise the development of new regulations and guidelines that formalize the management of sinks, showers, and sewage systems in hospitals and should prescribe a number of specific requirements. These new guidelines should be in line with guidelines that aim to prevent health care–acquired legionnaires’ disease. We recommend further studies to confirm the spread of *P aeruginosa* but also trials to determine clinical and environmental benefits of plumbing (shower and sink) changes or changing technology such as novel sink drains that reduce splashing.

## Notes

Supplement. **eTable.** Overview of Environmental Sampling
