# Supplementary material for: Risk Assessment After a Severe Hospital-Acquired Infection Associated With Carbapenemase-Producing Pseudomonas aeruginosa
Source: JAMA Netw Open. 2019 Feb 15;2(2):e187665. doi: 10.1001/jamanetworkopen.2018.7665 (PMC6484879; doi:10.1001/jamanetworkopen.2018.7665)
Supplement: Supplement. — eTable. Overview of Environmental Sampling [file jamanetwopen-2-e187665-s001.pdf]

Supplementary Online Content

Hopman J, Meijer C, Kenters N, et al. Risk assessment after a severe hospital-acquired infection associated with carbapenemase-producing *Pseudomonas aeruginosa*. *JAMA Netw Open.* 2019;2(2):e187665. doi:10.1001/jamanetworkopen.2018.7665

eTable. Overview of Environmental Sampling

This supplementary material has been provided by the authors to give readers additional information about their work.

© 2019 Hopman J et al. *JAMA Network Open.*

eTable. Overview of Environmental Sampling

Room nr.	Environmental sample	Shower drain	Air sample t=0 min	Air sample t=15 min
50	31-1-2018	N/A	N/A	N/A
	5-2-2018	Other gram-negative bacilli (GNB)	N/A	N/A
	12-2-2018	N/A	N/A	N/A
	16-2-2018	***Pseudomonas aeruginosa*** **VIM** Other GNB	NG	NG
	23-2-2018	***Pseudomonas aeruginosa*** **VIM** Other GNB	NG	Other GNB
51	31-1-2018	N/A	N/A	N/A
	5-2-2018	***Pseudomonas aeruginosa*** **VIM** Other GNB	N/A	N/A
	12-2-2018	***Pseudomonas aeruginosa*** **VIM**	Other GNB	Other GNB
	16-2-2018	Other GNB	NG	NG
	23-2-2018	Other GNB	NG	NG
52	31-1-2018	N/A	N/A	N/A
	5-2-2018	Other GNB	N/A	N/A
	12-2-2018	Other GNB	Other GNB	***Pseudomonas aeruginosa*** **VIM** Other GNB
	16-2-2018	***Pseudomonas aeruginosa*** **VIM** Other GNB	Other GNB	NG
	23-2-2018	***Pseudomonas aeruginosa*** **VIM**	NG	NG
53	31-1-2018	N/A	N/A	N/A
	5-2-2018	Other GNB	N/A	N/A
	12-2-2018	N/A	N/A	N/A
	16-2-2018	***Pseudomonas aeruginosa*** **VIM** Other GNB	Other GNB	NG
	23-2-2018	***Pseudomonas aeruginosa*** **VIM** Other GNB	NG	NG
54	31-1-2018	Other GNB	N/A	N/A
	5-2-2018	Other GNB	N/A	N/A
	12-2-2018	N/A	N/A	N/A
	16-2-2018	***Pseudomonas aeruginosa*** **VIM** Other GNB	NG	NG

© 2019 Hopman J et al. *JAMA Network Open.*

	23-2-2018	***Pseudomonas aeruginosa*** **VIM** Other GNB	NG	NG
55	31-1-2018	N/A	N/A	N/A
	5-2-2018	Other GNB	N/A	N/A
	12-2-2018	N/A	N/A	N/A
	16-2-2018	***Pseudomonas aeruginosa*** **VIM** Other GNB	NG	Other GNB
	23-2-2018	Other GNR	NG	NG
56	31-1-2018	***Pseudomonas aeruginosa*** **VIM** Other GNB	N/A	N/A
	5-2-2018	***Pseudomonas aeruginosa*** **VIM** Other GNB	N/A	N/A
	12-2-2018	***Pseudomonas aeruginosa*** **VIM**	NG	NG
	16-2-2018	***Pseudomonas aeruginosa*** **VIM** Other GNB	NG	Other GNB
	23-2-2018	Other GNB	NG	Other GNB
57	31-1-2018	N/A	N/A	N/A
	5-2-2018	***Pseudomonas aeruginosa*** **VIM**	N/A	N/A
	12-2-2018	***Pseudomonas aeruginosa*** **VIM** Other GNB	NG	NG
	16-2-2018	***Pseudomonas aeruginosa*** **VIM** Other GNB	NG	NG
	23-2-2018	Other GNB	NG	Other GNB
58	31-1-2018	N/A	N/A	N/A
	5-2-2018	Other GNB	N/A	N/A
	12-2-2018	N/A	N/A	N/A
	16-2-2018	Other GNB	NG	NG
	23-2-2018	Other GNB	Other GNB	Other GNB
59	31-1-2018	N/A	N/A	N/A
	5-2-2018	Other GNB	N/A	N/A
	12-2-2018	N/A	N/A	N/A
	16-2-2018	Other GNB	Other GNB	NG
	23-2-2018	Other GNB	NG	NG
60	31-1-2018	N/A	N/A	N/A
	5-2-2018	Other GNB	N/A	N/A

© 2019 Hopman J et al. *JAMA Network Open.*

	12-2-2018	N/A	N/A	N/A
	16-2-2018	Other GNB	NG	NG
	23-2-2018	Other GNB	NG	NG
*after cleaning and disinfection intervention				

© 2019 Hopman J et al. *JAMA Network Open.*
